# Melatonin prevents cyclophosphamide-induced primordial follicle loss by inhibiting ovarian granulosa cell apoptosis and maintaining AMH expression

**DOI:** 10.3389/fendo.2022.895095

**Published:** 2022-08-03

**Authors:** Juan Feng, Wen-Wen Ma, Hui-Xia Li, Xiu-Ying Pei, Shou-Long Deng, Hua Jia, Wen-Zhi Ma

**Affiliations:** ^1^ Key Laboratory of Fertility Preservation and Maintenance of Ministry of Education, and Key Laboratory of Reproduction and Genetics of Ningxia Hui Autonomous Region, School of Basic Medical Science, Ningxia Medical University, Yinchuan, China; ^2^ NHC Key Laboratory of Human Disease Comparative Medicine, Institute of Laboratory Animal Sciences, Chinese Academy of Medical Sciences and Comparative Medicine Center, Peking Union Medical College, Beijing, China

**Keywords:** melatonin, cyclophosphamide, primordial follicle, anti-Mullerian hormone, granulosa cell, apoptosis

## Abstract

Cyclophosphaty -45mide (Cyc) chemotherapy in young female cancer patients is associated with an increased risk of premature ovarian insufficiency (POI). This study was designed to investigate the protective role of melatonin (Mel) as an adjuvant against Cyc-induced POI. Female mice received a single intraperitoneal (i.p.) dose of Cyc (75 mg/kg). Mel protection was achieved in mice after i.p. injection of melatonin (50 mg/kg) every 24 h for four consecutive days prior to chemotherapy initiation and for 14 additional days. Ovarian reserve testing, hormonal assays for follicle-stimulating hormone, luteinizing hormone, and anti-Müllerian hormone (AMH), assessment of the oxidative stress status, and measurement of the relative expression of genes in PTEN/AKT/FOXO3a and mitochondrial apoptosis pathways were performed. The results showed that treatment with 50 mg/kg Mel significantly prevented Cyc-induced over-activation of primordial follicles by maintaining the plasma level of AMH and subsequently preventing litter size reduction in mice treated with Cyc chemotherapy. Importantly, Mel treatment significantly prevented ovarian granulosa cell loss by inhibiting the mitochondrial apoptotic pathway. Identifying the protective actions of Mel against Cyc-induced primordial follicle loss has important implications for fertility maintenance in young cancer patients undergoing chemotherapy.

## Introduction

With the increasing number of cancer survivors among children and adolescents, the issue of fertility preservation has assumed greater importance. All young patients with cancer or leukemia should have their fertility prognosis discussed before treatment initiation. The commonly used method of fertility preservation in female children is the freezing of ovarian tissue or unfertilized oocytes. These fertility preservation methods among children and adolescents are in the experimental stage, and none provide 100% effectiveness (1–3). For young female cancer patients who require immediate chemotherapy, finding an adjuvant to protect their ovaries during chemotherapy is one of the best options available to preserve fertility.

There are two main pathways for premature ovarian failure (POF) caused by the depletion of primordial follicle reserves after chemotherapy (4, 5); one is the direct toxic effect of the agent on follicular oocytes and granulosa cells, and the other is indirect over-activation of primordial follicles through damage to growing follicles (6, 7). The second pathway is the result of loss of negative feedback regulation, which inhibits primordial follicle activation (8, 9). Cyclophosphamide (Cyc), a commonly used chemotherapy, has a wide range of effects as a broad-spectrum antineoplastic drug (9, 10). Cyc induces apoptosis of granulosa cells in growing follicles by activating Bax and the mitochondrial apoptosis pathway (11). Granulosa cells of growing follicles proliferate rapidly and are highly sensitive to chemotherapeutic drugs (12).

Anti-Müllerian hormone (AMH) is a member of the transforming growth factor-β superfamily, which is mainly produced by granulosa cells in secondary and early antral follicles (6, 12–14). AMH is an important negative regulator of primordial follicle recruitment; it inhibits the activation of primordial follicles and reduces the sensitivity of antral follicles to follicle-stimulating hormone (FSH) during the recruitment cycle, thus maintaining a certain number of primordial follicles and growing follicles in the ovary (15). Cyc-induced apoptosis of granulosa cells in growing follicles decreases the level of AMH, reduces the inhibitory effect of AMH on primordial follicle recruitment, and enhances the sensitivity of antral follicles to FSH during cycle recruitment, resulting in a further decrease in AMH levels and indirect over-activation of primordial follicle reserves (12, 16).

Melatonin (Mel) attenuates Cyc-induced loss of primordial follicle loss and is produced by the pineal gland, ovaries, and placenta (17–20). Previously, Mel was found to reduce POF in mice caused by oxidative stress through the sirt1 signaling pathway (21). Mel also has anticancer effects due to its activation of the p53 and p21 signaling pathways, thereby inhibiting cancer cell growth and downregulating the vascular endothelial growth factor receptor to reduce angiogenesis (22, 23). Mel is an ideal adjuvant for chemotherapy because it neutralizes superoxide anions, hydrogen peroxide, and other oxygen-based free radicals.

Studies have shown that Mel protects against damage to the primordial follicular pool caused by cisplatin chemotherapy by inhibiting the PTEN/AKT/FOXO3a signaling pathway (6). However, the mechanism by which Mel prevents ovarian POF after Cyc chemotherapy remains unclear. Here, we investigated the mechanism by which Mel prevents Cyc-induced ovarian POF. We verified that Mel administration successfully rescued Cyc-induced primordial follicle loss by inhibiting ovarian granulosa cell apoptosis and maintaining AMH expression.

## Materials and methods

### Animals experiments

**Figure 1:**
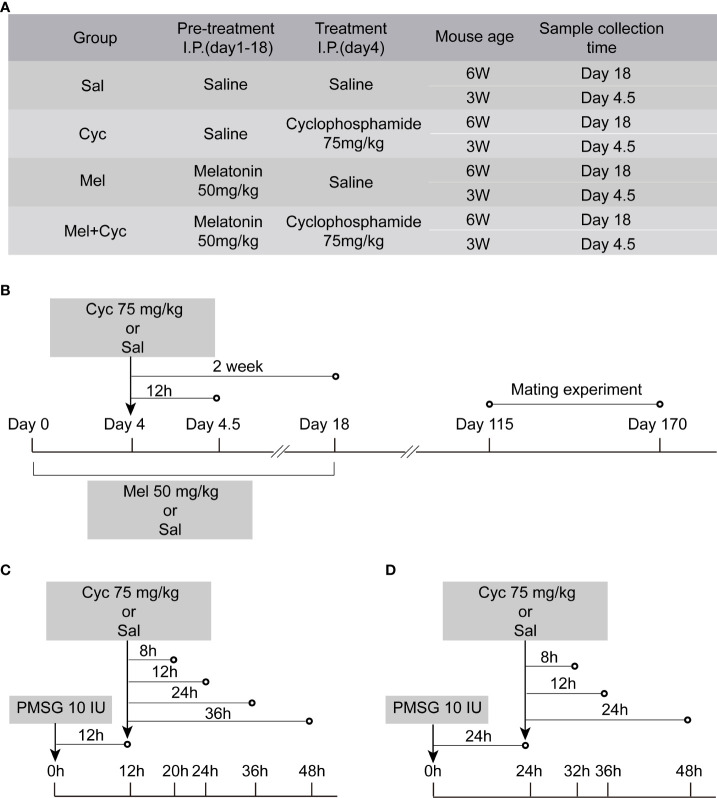
Graphic illustration of experimental schedule. **(A)** Mice were randomly divided into four groups. **(B)** Six or three-week-old ICR mice were pre-treated with i.p. injections of Mel or Sal once daily (at 24 h intervals) for four consecutive days before achieving Cyc injection, and for 14 additional days during chemotherapy treatment. On Day 4, mice were injected with Sal or Cyc (75 mg/kg). Blood and/or ovaries were collected 12 h or two weeks after Cyc injection. Fertility assessment was performed 115 days after Cyc injection. **(C)** Three-week-old prepubertal female mice were injected i.p. with 75 mg/kg body weight Cyc 12 h after injection of PMSG. Mice were sacrificed 8, 12, 24, or 36 h after chemotherapy treatment and their ovaries were excised (n = 4). **(D)** Prepubertal female mice were injected i.p. with 75 mg/kg body weight Cyc 24 h after injection of PMSG. Mice were sacrificed 12, 24, or 36 h after chemotherapy treatment and their ovaries were excised.

Six-week-old and three-week-old female Institute of Cancer Research (ICR) mice were purchased from the Experimental Animal Center of the Ningxia Medical University. Animals were fed *ad libitum* and had free access to food and water. They were housed under conditions of constant temperature (21 ± 2°C) and a 12-hr light/dark cycle. All mice were acclimated for three days before the experiment. The experiment was approved by the Animal Care and Use Committee of Ningxia Medical University. Six-week-old and three-week-old female mice were randomly divided into four experimental groups: saline (Sal, control), Cyc, Mel, and Mel + Cyc. There were 25 six-week-old and 48 three-week-old female mice in each group. Mel protection was achieved in mice after intraperitoneal (i.p.) injection of Mel (50 mg/kg body weight; Sigma, St. Louis, MO, USA) or Sal, administered every 24 h for four consecutive days prior to chemotherapy initiation and for 14 additional days to maintain high levels during chemotherapeutic treatment. After the initial 4-day Mel treatment period, mice were injected i.p. with Cyc (75 mg/kg, Sigma) or Sal. The standard dosage of Cyc was selected based on previous studies showing ovarian damage (16, 24). Eighteen six-week-old female mice in each group were euthanized and blood samples and ovaries were collected 14 days after Cyc injection. Seven six-week-old female mice in each group were used for the mating experiment 115 days after the Cyc injection. For three-week-old female mice, ovaries were collected for the evaluation of apoptosis 12 h after Cyc injection (**Figure 1A**). Thereafter, maintenance of the primordial follicle pool was evaluated by fertility assessments from 115 to 170 days after Cyc injection (**Figure 1B**). To avoid a false positive of granulosa cell apoptosis during physiological follicular atresia in adult mice, untreated prepubertal three-week-old female mice aged were used, and their follicular development was activated by injection of 10 IU of pregnant mare’s serum gonadotropin (PMSG). Mice were injected i.p. with 75 mg/kg body weight Cyc 12 or 24 h after the injection of PMSG. They were sacrificed at 8, 12, 24, and 36 h after chemotherapy (the longest time was no more than 48 h after PMSG injection), and their ovaries were excised for apoptosis detection (**Figures 1C, D**). To study the function and mechanism of Mel intervention on apoptosis of granulosa cells in prepubertal three-week-old mice, 12 h was selected as the interval after Cyc injection in the subsequent experiments (**Figure 1B**).

### Histology and follicle counts

Follicle counting was performed according to previously published methods (25, 26). Briefly, ovaries were fixed in 4% paraformaldehyde overnight, embedded in paraffin, and cut into 5-μm serial sections. The total number of follicles in each ovary was estimated by counting the number of follicles in every fifth HE-stained section and applying a five-fold correction factor. Follicles were divided into five stages: primordial, primary, secondary, antral, and atretic follicles. Only the follicles with apparent oocyte nuclei were counted. Oocytes surrounded by squamous granulosa cells were classified as primordial follicles. The primary follicles were oocytes surrounded by a layer of cubic granulosa cells. Secondary follicles had two or more layers of cubic granular cells but lacked a lumen. Antral follicles have several layers of granular cells and lumens (27). Atretic follicles had aberrant oocytes and multiple layers of pycnotic granulosa cells.

### Immunohistochemistry

Paraffin-embedded sections of mouse ovaries were dewaxed, hydrated, and sealed with 5% normal goat serum after antigen repair. The sections were incubated overnight in anti-Nobox (1:500, Bioss, Woburn, MA, USA; Nobox is an oocyte-specific homeobox gene that plays a critical role in early folliculogenesis (28) and anti-AMH (1:2000, Abcam, Cambridge, UK) at 4°C, and then biotinylated rabbit anti-mouse antibody was added. Immunoreactivity was detected by indirect immunoperoxidase staining (Vector Labs, Newark, CA, USA) with DAB. Images were obtained under a microscope.

### Fertility assessment

At 115 days after Cyc treatment, sexually mature ICR female mice were paired with healthy male mice in a 2:1 ratio (26, 29). All male mice used for mating were approximately 12 weeks old and confirmed to be fertile. Female mice were kept with male mice until vaginal plugs were observed in the morning (n = 7). By the fifteenth day after mating, mated female mice that were judged to be pregnant were isolated. After delivery, postnatal mice and mice that failed to conceive were reintroduced to the male for the next round of mating.

### Measurement of serum AMH, FSH, and luteinizing hormone

The six-week-old female mice were anesthetized, and blood was collected from the orbital vein behind the eyeball 14 days after chemotherapy. Sera were then separated by centrifugation (4000 × g, 15 min, 4°C) and frozen at -80°C. Serum AMH concentration was measured using a mouse AMH ELISA kit (Elabscience Biotechnology, Bethesda, MD, USA). The serum concentration of FSH was measured using a mouse follicle-stimulating hormone ELISA kit (Jianglai Biotechnology, Shanghai, China). The serum luteinizing hormone (LH) concentration was measured using a mouse luteinizing hormone ELISA kit (Jianglai Biotechnology, Shanghai, China). All measurements were performed in accordance with the manufacturer’s instructions.

### Measurement of malondialdehyde levels and activities of superoxide dismutase and catalase

Fresh tissue from six-week-old female mice were washed with ice-cold phosphate buffered saline (PBS) solution and weighed. After the weights were recorded, homogenization was immediately performed using a tissue homogenizer on ice and centrifuged. The supernatants were used for the measurements. Malondialdehyde (MDA), superoxide dismutase (SOD) and catalase (CAT) assays were performed using a spectrophotometer (Jianglai Biotechnology, Shanghai, China). The analysis was performed in accordance with the manufacturer’s instructions.

### Evaluation of apoptosis

After deparaffinization of three-week-old female mouse ovary slides, the TUNEL BrightGreen Apoptosis Detection Kit Vazyme Code (Vazyme A112-02) was used to perform TUNEL analysis according to the manufacturer’s instructions. During apoptosis, intracellular endonucleases are activated, chromatin DNA is specifically cleaved between nucleosomes, and DNA is degraded into 180–200 bp or integer multiple fragments. Nucleotidyl Transferase binds FITC-12-dUTP to the 3’ -hydroxyl (3’ -OH) end of the DNA molecule break, which can bind FITC-12-dUTP under the action of terminal deoxynucleotidyl transferase. The FITC-12-dUTP-labeled broken DNA can be directly observed with a fluorescence microscope (green represents apoptosis) to reflect apoptosis levels.

### Western blot analysis

The ovarian tissues of mice in each group were extracted with RIPA lysis buffer, and the protein concentration was determined using the bicinchoninic acid assay. The total protein from each group was added to the sample buffer for PAGE. Total proteins were separated by electrophoresis on 10–12.5% SDS-PAGE gels. Cytoplasmic and mitochondrial proteins were prepared using a cytoplasmic and mitochondrial protein extraction kit purchased from Sangon Biotech (Shanghai, China) (30). Cytoplasmic and mitochondrial protein extraction was performed according to the manufacturer’s instructions. Briefly, the cytoplasm and mitochondria were placed directly in the sample buffer and boiled for 10 min. Protein samples were separated by electrophoresis on 15% SDS-PAGE gels. After electrophoresis, steps such as closure, membrane transfer, hybridization, and exposure, were performed. The primary antibodies included anti-p-PTEN antibody (1:1000, Affinity Biosciences, Cincinnati, OH, USA), anti-PTEN antibody (1:1000, Affinity, Inc.), anti-p-FOXO3a antibody (1:5000, Abcam), anti-FOXO3a antibody (1:1000, Cell Signaling Technology, Danvers, MA, USA), anti-active-Caspase3 (1:5000, Abcam), anti-Caspase-3 antibody (1:2000, Abcam), anti-Bax antibody (1:1000, Cell Signaling Technology), anti-Bcl-2 antibody (1:1000, Cell Signaling Technology), anti-cytochrome C antibody (1:5000, Abcam), anti-VDAC1 antibody (1:5000, Abcam), anti-β-actin antibody (1:5000, Proteintech, Rosemont, IL, USA), anti-GAPDH (1:2000, Bioss), and β-tubulin (1:3000, Affinity Biosciences). The appropriate horseradish peroxidase-conjugated secondary antibodies were diluted 1:20000 in 1× PBS and added to the membranes for 1 h at room temperature. The protein bands were visualized using enhanced chemiluminescence reagent. ImageJ software was used to analyze the band intensities. β-actin, β-tubulin, and VDAC1 were used as internal controls.

### Statistical analysis

All analyses were performed using Prism 7 software (GraphPad, San Diego, CA, USA). When the distribution of data was not normal, a Mann–Whitney U test was used for analysis. Statistical differences of follicle number, litter size, hormone levels and western blot results in four experimental groups were assessed by one-way ANOVA for multiple comparisons followed by Bonferroni *post hoc* analysis. A P-value <0.05 was considered statistically significant.

## Results

### Mel prevents Cyc-induced primordial follicle loss

**Figure 2:**
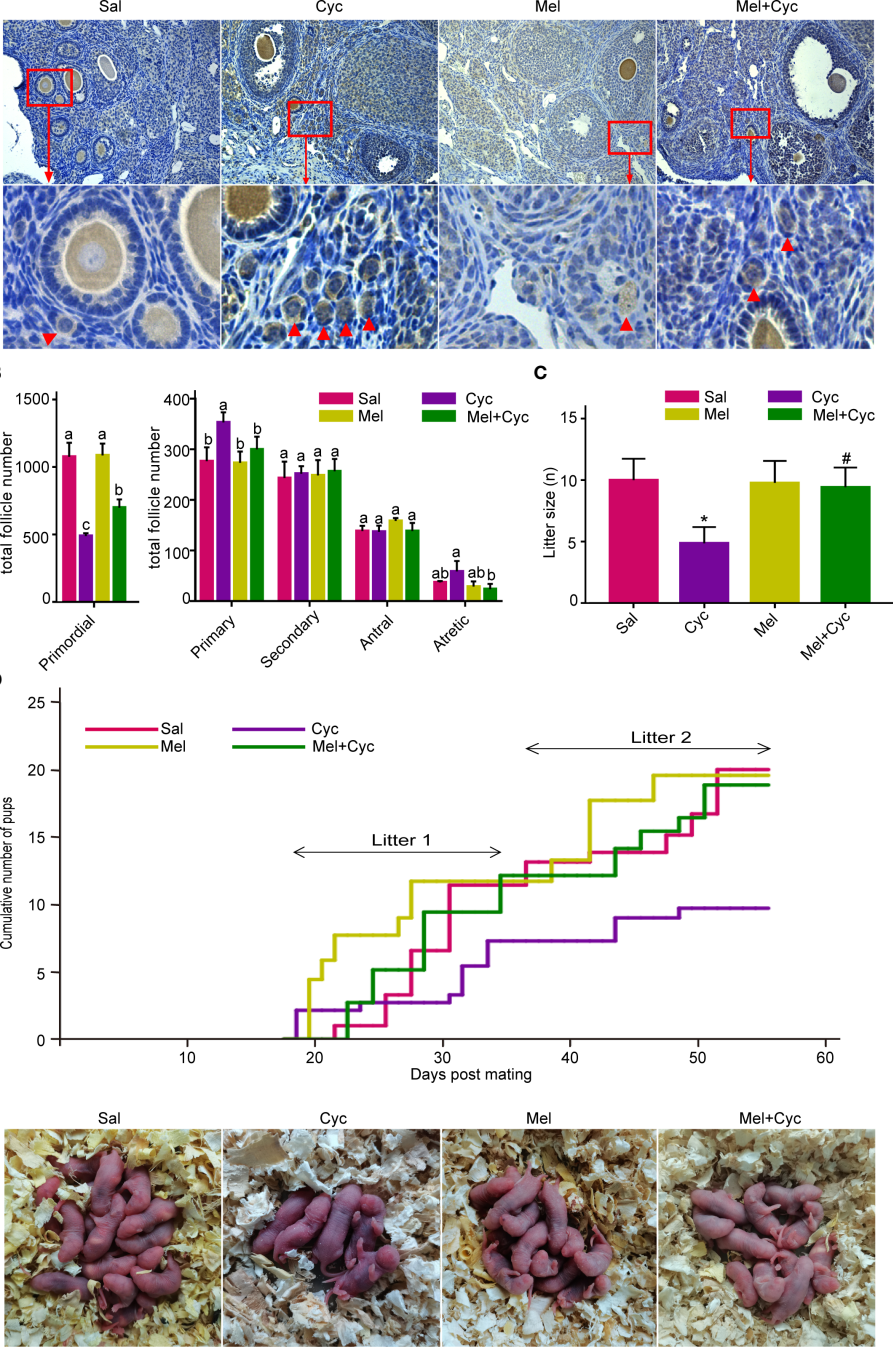
Mel prevents Cyc-induced primordial follicle loss. There were 25 six-week-old female mice in each group, of which 18 were sacrificed to obtain the ovary and blood samples, and seven mice were used for the mating experiment. **(A)** Oocytes in mouse ovaries were detected using an Nobox antibody (n = 6). Red arrow indicates primordial follicles. Bar = 200 μm. **(B)** Follicle count of whole ovarian tissue in control and treatment group mice 14 days after Cyc chemotherapy (n = 6). Mel prevented Cyc-induced dormant primordial follicle loss and suppressed the activation of primordial follicles into primary follicles. ^a,b,c,d^ Values with different letters in the same type of follicles were significantly different from each other. **(C)** Cyc treatment reduced the mean litter size in mice and Mel reversed this effect (n = 7). *P < 0.05 compared with Sal group. ^#^ *P* < 0.05 compared with Cyc group. **(D)** Cumulative pup number of each mouse in control and treatment groups at Day 55 of mating (n = 7). **(E)** Representative litters from control and treatment groups.

To determine whether Cyc activates primordial follicles, Mel has a protective function against Cyc-induced primordial follicle loss. Follicle number and litter size in the mice treated with or without Mel were counted after chemotherapy. Oocytes were stained with Nobox antibody to determine the location of primordial follicles in mouse ovarian tissue. The primordial follicles were labeled (**Figure 2A**). The number of primordial follicles, primary, secondary, antral, and atretic follicles were counted. After Cyc treatment alone, the number of primordial follicles in the Cyc group was significantly reduced compared to that in the Sal group (*P* < 0.05). In addition, the number of primordial follicles in mice treated with Mel during Cyc chemotherapy (Mel + Cyc group) was significantly higher than that in Cyc-treated mice (Cyc group) (*P* < 0.05).

Compared to the Sal (control) group, Cyc treatment also increased the number of primary follicles, which was significantly reduced by Mel treatment (**Figure 2B**). Compared to the control group, the average litter size was significantly lower after Cyc chemotherapy on Day 55 (from 115 days after chemotherapy to 170 days after chemotherapy); however, Mel co-treatment significantly increased the average litter size after Cyc treatment (**Figures 2C, E**). The cumulative pup number of each mouse in the Cyc group was lower than that in the Sal and Mel groups, and Mel co-treatment increased the cumulative number of pups in the Mel + Cyc group (**Figure 2D**). Overall, Mel treatment prevented Cyc-induced dormant primordial follicle loss and suppressed the conversion of primordial follicles to primary follicles.

### Mel significantly improves expression of AMH after Cyc chemotherapy

**Figure 3:**
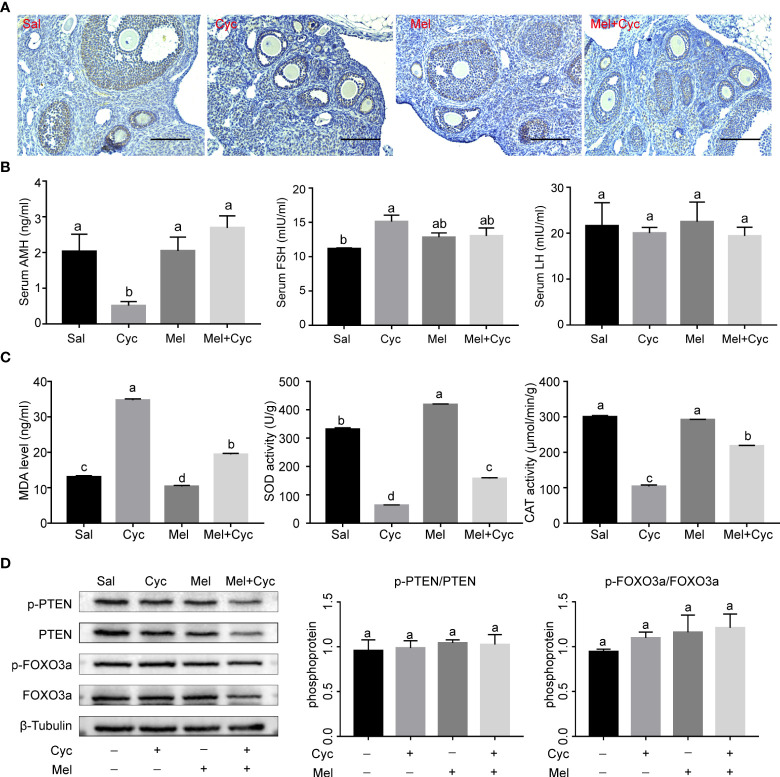
Effect of Mel on expression of AMH and oxidative stress in ovaries of six-week-old female mice 14 days after Cyc chemotherapy (Day 18). **(A)** AMH was expressed in granulosa cells of secondary and early antral follicles in Sal, Cyc, Mel, and Mel + Cyc groups (n = 6). Bar = 200 μm. **(B)** Cyc combined with Mel therapy prevented the decrease of serum AMH and LH levels but there was no significant change in serum FSH levels (n = 6). **(C)** Biochemical analysis of the ovarian CAT, SOD activities, and MDA levels (n = 6). **(D)** Phosphorylation levels of PTEN and FOXO3a did not change significantly after treatment with Cyc and Mel (n = 6). ^a,b^ Values with different letters are significantly different from each other.

To determine whether Cyc chemotherapy induced primordial follicle loss through indirect overactivation of primordial follicles and activation of the PTEN/AKT/FOXO3a pathway, FOXO3a phosphorylation levels and serum AMH concentrations were measured. The results revealed that serum AMH and LH levels in the Cyc group decreased significantly 14 days after Cyc chemotherapy, but combination therapy with Mel significantly prevented the Cyc-induced decrease in serum AMH and LH levels in the Mel + Cyc group (*P* < 0.05). Serum FSH levels in the Cyc group were not significantly different from those in the Mel + Cyc group (*P* > 0.05) (**Figures 3A, B**). Biochemical analysis of ovarian tissue for antioxidant enzymes showed a significant increase in MDA in the Cyc group after Cyc chemotherapy for 14 days, but combined therapy with Mel significantly prevented the Cyc-induced increase in MDA levels in the Mel + Cyc group (*P* < 0.05). Tissue SOD and CAT activities were significantly higher in the Mel + Cyc group than in the Cyc group and lower than those in the Sal and Mel groups in the ovarian homogenates (*P* < 0.05) (**Figure 3C**). However, no significant changes were observed in the phosphorylation levels of FOXO3a and PTEN (**Figure 3D**). These results indicate that Cyc chemotherapy over activates primordial follicles through an AMH-mediated indirect pathway, whereas the FOXO3a-mediated pathway does not play a role in this process.

### Co-treatment with Mel prevented apoptosis in granulosa cells

**Figure 4:**
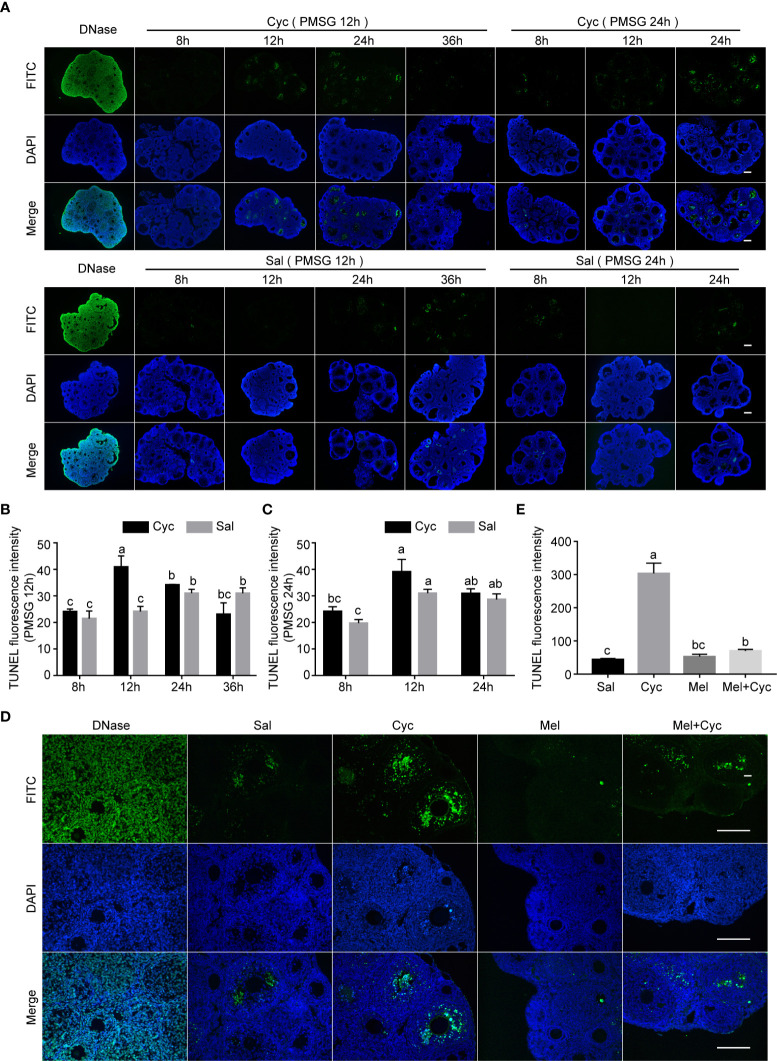
Cyc induced apoptosis of granulosa cells in growing follicles and Mel prevented this apoptosis. **(A)** Three-week-old ICR mice were injected i.p. with Cyc 12 or 24 h after injection of PMSG, and apoptosis in ovarian tissues was detected by TUNEL kit at 8, 12, 24, and 36 h after Cyc injection. Regardless of whether Cyc was injected 12 or 24 h after PMSG injection, apoptotic peak of granulosa cells in ovary appeared 12 h after Cyc intervention, which was not related to the time of PMSG administration. Mice in control group were injected with Sal instead of Cyc, and the apoptotic peak of granulosa cells appeared 48 h (PMSG 12 h + Sal 36 h, or PMSG 24 h + Sal 24 h) after injection of PMSG. DNase I treatment was used for positive control of TUNEL assay. There were four three-week-old female mice in each group; Bar = 200 μm. **(B)** TUNEL fluorescence intensity in ovaries of 3-week-old ICR mice 8, 12, 24 and 36 hours after Cyc injection. The mice received Cyc chemotherapy 12 hours after injection of PMSG. **(C)** TUNEL fluorescence intensity in ovaries of 3-week-old ICR mice 8, 12 and 24 hours after Cyc injection. The mice received Cyc chemotherapy 24 hours after injection of PMSG. **(D)** Mel prevented apoptosis of granulosa cells 12 h after Cyc chemotherapy. There were four three-week-old female mice in each group; Bar = 200 μm. **(E)** TUNEL fluorescence intensity in ovaries of 3-week-old ICR mice in Sal, Cyc, Mel and Mel+Cyc groups 12 hours after Cyc injection.

To analyze whether Mel maintained AMH expression by inhibiting ovarian granulosa cell apoptosis in growing follicles, we measured the incidence of apoptosis of granulosa cells in prepubertal three-week-old female mice after Cyc and/or Mel treatment. Cyc treatment promoted apoptosis of ovarian granulosa cells and the apoptotic peak of granulosa cells was not associated with the injection of PMSG 12 or 24 h in advance but occurred 12 h after Cyc injection (**Figures 4A–C**). Therefore, we selected 12 h after Cyc injection as the time point to study the effect of Mel intervention on apoptosis of granulosa cells and found that Mel significantly reduced the number of TUNEL-positive apoptotic granulosa cells in growing follicles after Cyc chemotherapy (**Figures 4D, E**).

### Mel inhibits Cyc-induced apoptosis of granulosa cells through mitochondrial apoptosis pathway

**Figure 5:**
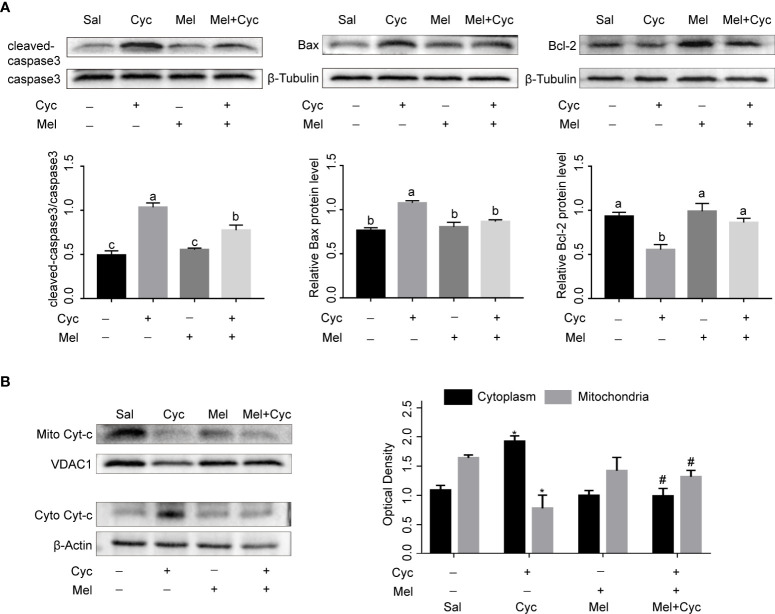
Mel inhibits Cyc-induced apoptosis of granulosa cells through mitochondrial apoptosis pathway. **(A)** Western blot analysis was performed on cleaved-caspase3, caspase3, Bax, and Bcl-2 in the ovaries of three-week-old ICR mice 12 h after Cyc (75 mg/kg) treatment with or without Mel (n = 6). ^a,b,c^ Bars with different letters are significantly different in each group (*P < 0.05*). **(B)** Western blot analysis was performed on mitochondrial Cyt-c (mito Cyt-c) and cytoplasm Cyt-c (cyto Cyt-c) in the ovaries of three-week-old ICR mice 12 h after Cyc (75 mg/kg) treatment with or without Mel (n = 6). *P < 0.05 compared with Sal group. ^#^ *P* < 0.05 compared with Cyc group.

To further investigate the inhibitory effect and mechanism of Mel on Cyc-induced apoptosis of granulosa cells, we measured the expression of caspase3, Bax, Bcl-2, and Cyt-C proteins. The results showed that Cyc significantly increased the expression of cleaved-caspase3, Bax, and cytoplasmic Cyt-c and decreased the expression of Bcl-2 in ovaries (*P* < 0.05). Co-treatment with Mel significantly reduced Cyc-induced up-regulated expression of cleaved-caspase3, Bax, and cytoplasmic Cyt-c, and increased the Cyc-induced down-regulated expression of Bcl-2 in the ovaries (*P* < 0.05) (**Figures 5A, B**). These findings suggest that the mitochondrial apoptosis pathway is involved in Cyc-induced apoptosis in granulosa cells, and Mel inhibits the apoptosis pathway.

## Discussion

In the present study, we demonstrated that Cyc treatment promoted apoptosis of ovarian granulosa cells through the mitochondrial apoptotic pathway. Apoptosis of granulosa cells reduces AMH secretion and depletes the dormant follicle pool in mouse ovaries through indirect over-activation of primordial follicles. Furthermore, we found that Mel co-treatment significantly prevented Cyc-induced apoptosis of ovarian granulosa cells, which maintained AMH expression in granulosa cells during chemotherapy, and prevented Cyc-induced primordial follicle loss in mouse ovaries.

Cyc is a chemotherapeutic drug that is highly toxic to ovaries. It is a prodrug that is activated by cytochrome p450 enzymes to produce its active metabolites. The latter is responsible for ovarian toxicity. Cyc does not induce degeneration of primordial follicles. Instead, Cyc induces apoptosis of actively growing follicles and activates primordial follicles to primary follicles, leading to the depletion of primordial follicles (8). Cyc induces apoptosis through two pathways, one of which involves Cyc inhibition of the synthesis of DNA by cross-linking with cell DNA in all stages of the cell cycle, causing cellular apoptosis (10, 31). The other pathway involves the reduction of mitochondrial transmembrane potential caused by Cyc and accumulation of Cyt-c in the cytosol of rat granulosa cells, which leads to the activation of the caspase family and apoptosis (11). Cyc also upregulates the expression of Bax protein and downregulates the expression of Bcl-2 which causes the reduction of mitochondrial transmembrane potential, causing activation of the apoptotic cascade (11). Our results suggest that Cyc stimulates apoptosis in growing follicular granule cells through a mitochondria-dependent pathway in mice. The apoptosis of granulosa cells reduces AMH secretion and depletes the dormant follicle pool in mouse ovaries through indirect over-activation of primordial follicles. However, the addition of Mel inhibited apoptosis of granulosa cells induced by Cyc and maintained AMH expression in the ovaries.

Physiologically, apoptosis is an essential event for ovarian function, and the development of this organ is harmful when the ovary is exposed to Cyc (3, 32). The Cyc drug itself and its toxic metabolites also interfere with the intracellular antioxidant system, which plays an important function in detoxifying reactive oxygen species (33). SOD can transform superoxide anion into hydrogen peroxide, which plays a key role in antioxidant reaction (34, 35). CAT, another antioxidant enzyme, catalyzes only the decomposition of hydrogen peroxide into water and oxygen in the absence of an electron donor (34, 36). Biochemical measurements of MDA levels in tissues as a measure of lipid peroxidation have been used to assess oxidative stress and ovarian damage (35–37). The results of previous research indicate that Cyc decreases SOD and CAT activity, which means that the consumption of this antioxidant enzyme is increased by Cyc or its metabolites. In the present study, the parameters of oxidative stress, that is MDA, were significantly increased and the activities of SOD and CAT were significantly decreased in the ovaries of Cyc-treated mice, suggesting that Cyc treatment caused oxidative damage to lipids and proteins in this organ. The mouse ovaries in the Mel + Cyc group had significantly increased SOD and CAT activities and decreased MDA levels, suggesting that Mel protects against the adverse effects of Cyc.

Mel is a powerful free-radical scavenger and broad-spectrum antioxidant (38, 39). Mel has several important protective and regulatory functions. In addition to its role in regulating sleep, Mel also protects ovarian function, regulates immune function, and has anti-aging and anti-tumor (40–42). In the female reproductive system, Mel plays an important role in normal physiology and protects against ovarian pathologies (21, 43). Mel, a free radical scavenger in ovarian follicles, promotes oocyte maturation, embryo development, and luteinization of granulosa cells (44, 45). In the current study, we found that Mel plays an antioxidative role by reducing MDA content and increasing SOD and CAT activity. We also found that Mel ameliorates the effects of mitochondria-mediated apoptosis on ovarian tissue by inhibiting Bax expression, Cyt-c release from the mitochondria to the cytoplasm, caspase-3 activation, and induction of Bcl-2 expression. Mel protects ovarian granulosa cells from apoptosis induced by Cyc chemotherapy by inhibiting the mitochondrial apoptosis pathway, providing new evidence for Mel as an adjuvant chemotherapy agent.

AMH, which is secreted by the granulosa cells of growing follicles, represents a reliable biomedical marker of ovarian reserve and is informative for monitoring ovarian function after chemotherapy (46, 47). AMH level is an important indicator of ovarian function after chemotherapy. A rapid and significant decrease in AMH concentration has been observed in adult women after chemotherapy (48). Recent data also suggest that the AMH concentration before chemotherapy and the size of the drop and recovery of AMH during and after chemotherapy can be used to predict the degree of ovarian injury (49). Therefore, the use of adjuvant drugs to protect AMH levels during chemotherapy is particularly important for maintaining fertility in women treated with antitumor chemotherapy. When injected or overexpressed in mice concurrent with chemotherapy, AMH inhibits the activation of primordial follicles and prevents POF (12). Prevention of POF and protection of the ovarian follicle pool have become attractive avenues for improving the quality of life of female cancer patients receiving chemotherapy. Fertility preservation in female patients is important for their well-being after cancer survival. In the current study, Mel treatment significantly reversed the decrease in serum AMH levels, possibly because Mel intervention prevented Cyc-induced apoptosis of the follicular granulosa cells. The surviving granulosa cells secrete AMH and maintain normal serum AMH levels. AMH inhibits over-activation of primordial follicles through negative feedback regulation and prevents Cyc-induced primordial follicle loss.

Litter size is one of the methods used for the functional evaluation of the ovarian reserve after chemotherapy. primordial follicles generally require many weeks to develop into mature follicles in mice (50). In the present study, to ensure that mature oocytes were derived from primordial follicles after chemotherapy, mice were selected 115 days after Cyc treatment for two cycles of mating, and we found that Cyc significantly reduced the average litter size in mice. In the Mel intervention group, litter size increased significantly after chemotherapy. Apoptosis of granulosa cells induced by Cyc chemotherapy was reduced after intervention with Mel, and the surviving granulosa cells maintained the expression of AMH, thus inhibiting chemotherapy-induced hyperactivation of primordial follicles. In addition, a recent study showed that Cyc induces primordial follicle loss through the PTEN/AKT/FOXO3a signaling pathway (51); however, we showed here that Cyc had no effect on the PTEN/AKT/FOXO3a pathway. This may be related to the dose of Cyc, which was 200 mg/kg in the study performed by Barberino compared to 75 mg/kg used in the present study.

## Conclusion

**Figure 6:**
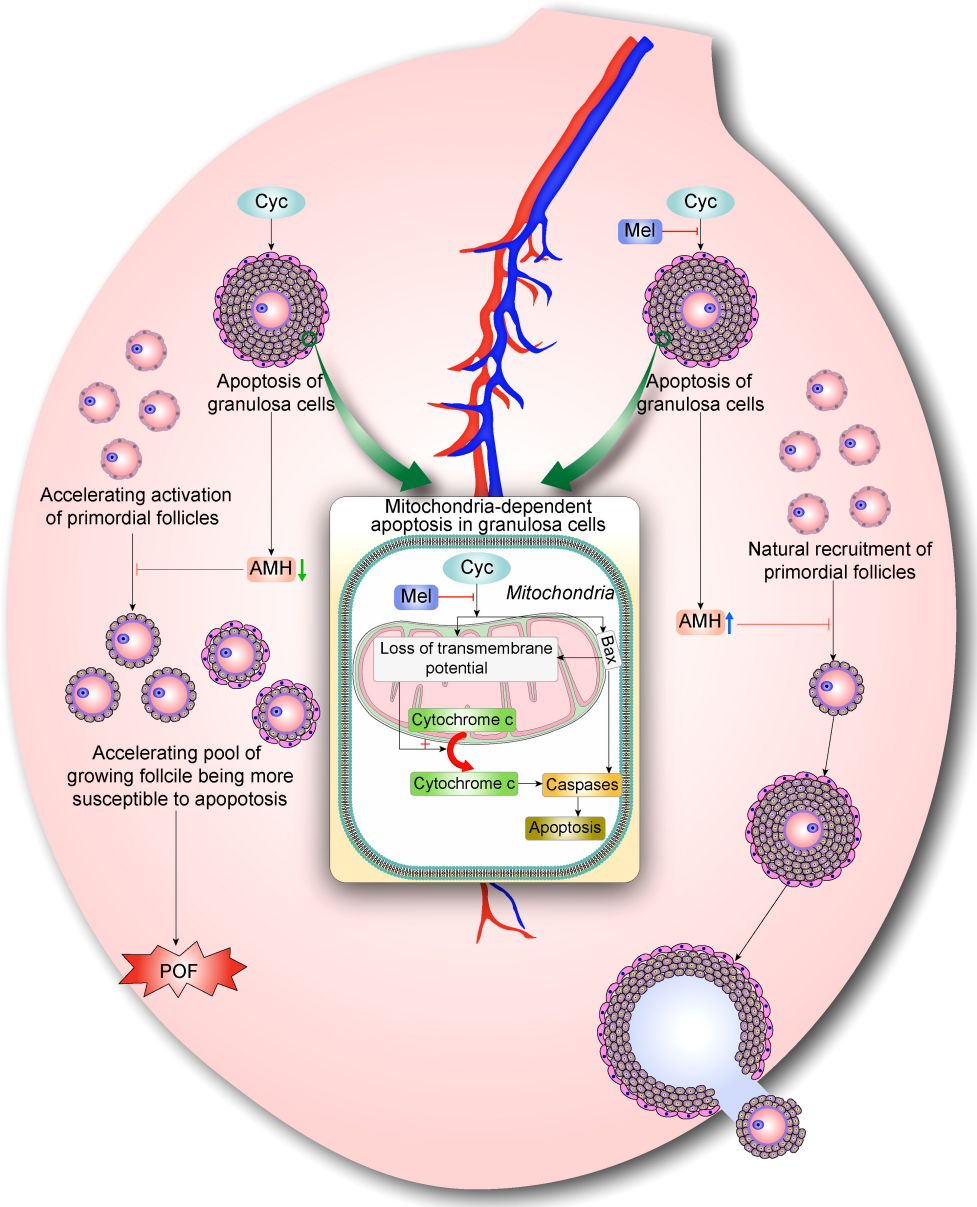
Mel prevents Cyc-induced over-activation of primordial follicles through inhibiting ovarian granulosa cell apoptosis and maintaining AMH expression. During normal follicle development, very few primordial follicles are selected and activated, whereas the vast majority of primordial follicles are maintained in a dormant state. AMH is mainly produced by granulosa cells in secondary follicles and early antral follicles. AMH can inhibit the activation of primordial follicles during recruitment and inhibit the sensitivity of antral follicles to FSH during recruitment cycle, thus maintaining AMH levels and selective activation of primordial follicle pool. Cyc exposure disturbs the balance of follicle activation by increasing apoptosis of AMH-secreting granulosa cells in growing follicles, resulting in further decrease of AMH levels and indirect over-activation of primordial follicle pool. Eventually, activated follicles undergo apoptosis and more primordial follicles are stimulated to become activated primary follicles, resulting in POF. With Mel co-treatment, activation of primordial follicles is reduced and apoptosis of AMH-secreting granulosa cells in growing follicles is decreased. Surviving granulosa cells maintained normal levels of AMH production, which regulates natural recruitment of primordial follicles and oogenesis. Hence, Mel restores the balance of follicle activation and returns ovaries to a healthy state.

In summary, our findings suggest that Mel prevents Cyc-induced over-activation of primordial follicles by inhibiting ovarian granulosa cell apoptosis and maintaining AMH expression. Cyc exposure disturbs the balance of follicle activation by increasing apoptosis of AMH-secreting granulosa cells in growing follicles, resulting in the further decrease of AMH levels and indirect over-activation of the primordial follicle pool. The activated follicles undergo apoptosis, and more primordial follicles are stimulated to become activated primary follicles, resulting in POF. However, Mel co-treatment reduced primordial follicle activation and apoptosis of AMH-secreting granulosa cells in growing follicles. Mel restored the balance of follicle activation and returned the ovary to a healthy state (**Figure 6**). These findings on the mechanisms underlying the protective effects of Mel against Cyc-induced primordial follicle loss have key implications for fertility maintenance in young cancer patients who undergo chemotherapy.

## Data availability statement

The original contributions presented in the study are included in the article/Supplementary Materials. Further inquiries can be directed to the corresponding authors.

## Ethics statement

The animal study was reviewed and approved by Animals Care and Use Committee of Ningxia Medical University.

## Author contributions

JF, W-WM, and H-XL were responsible for the experiments, data analysis and editing of the manuscript. X-YP, S-LD, and HJ participated in the design of the study and edited the manuscript. W-ZM contributed to the conception, supervision and editing of the manuscript. All authors read and approved the final manuscript.

## Funding

This work was supported by the Key Research and Development Program of Ningxia Hui Autonomous Region (2019BFG02007, 2021BEG02029), the National Natural Science Foundation of China (81860266), and the Natural Science Foundation of Ningxia Hui Autonomous Region (2022AAC03188).

## Acknowledgments

We sincerely thank Professor Russel J. Reiter (Department of Cell Systems and Anatomy, UT Health, San Antonio. TX) for kindly final editing and English corrections.

## Conflict of interest

The authors declare that the research was conducted in the absence of any commercial or financial relationships that could be construed as a potential conflict of interest.

## Publisher’s note

All claims expressed in this article are solely those of the authors and do not necessarily represent those of their affiliated organizations, or those of the publisher, the editors and the reviewers. Any product that may be evaluated in this article, or claim that may be made by its manufacturer, is not guaranteed or endorsed by the publisher.
